# The Effects of Various Restorative Techniques on the Fracture Resistance of Pulpotomized Permanent Premolars

**DOI:** 10.1155/2021/5590911

**Published:** 2021-05-08

**Authors:** Yasamin Ghahramani, Fereshteh Shafiei, Zahra Jowkar, Sepideh Kazemian

**Affiliations:** ^1^Department of Endodontics, School of Dentistry, Shiraz University of Medical Sciences, Shiraz, Iran; ^2^Oral and Dental Disease Research Center, Department of Operative Dentistry, School of Dentistry, Shiraz University of Medical Sciences, Shiraz, Iran; ^3^Department of Operative Dentistry, School of Dentistry, Shiraz University of Medical Sciences, Shiraz, Iran

## Abstract

**Aim:**

This study aimed to evaluate the effects of various restorative techniques on the fracture resistance of pulpotomized premolars with mesioocclusodistal (MOD) cavities treated with mineral trioxide aggregate (MTA) or calcium enriched mixture (CEM) cement.

**Materials and Methods:**

One hundred and eight sound extracted maxillary premolars were randomly assigned to nine experimental groups (*n* = 12). The teeth in group 1 did not receive any preparation. Class II MOD cavities were prepared in the other experimental groups. In groups 2, 4, 6, and 8, tooth-colored MTA was used for pulpotomy. In groups 3, 5, 7, and 9, CEM cement was used for pulpotomy. Groups 2 and 3 were left unrestored. Groups 4 and 5 were restored with amalgam. Groups 6 and 7 were restored with a conventional composite resin, and groups 8 and 9 were restored with bulk-fill giomers. Fracture resistance was measured, the fracture pattern of each specimen was assessed, and the results were statistically analyzed.

**Results:**

The fracture resistance of group 1 was significantly higher than those of the other groups (*p* < 0.05). The fracture resistance of group 2 (MTA + amalgam) was statistically lower than those of all experimental groups (*p* values < 0.05) except groups 3, 4, and 5 (*p* values > 0.05). No statistically significant differences were found between the groups restored with amalgam, conventional composite resin, and bulk-fill giomer (groups 4, 5, 6, 7, 8, and 9) (*p* values < 0.05). The highest rate of mode 1 fracture (restorable fracture) was observed in group 1 followed by groups 8 and 9.

**Conclusion:**

No significant differences were found among the fracture resistances of the restored teeth using various restorative techniques. Bulk-fill giomers followed by conventional composite resin were better able to prevent unfavorable fractures compared to amalgam. Therefore, they seem to be more reliable for the restoration of pulpotomized teeth with MOD cavities.

## 1. Introduction

An important biological and clinical challenge in immature permanent teeth is to preserve dental pulp [1]. If a deep caries lesion results in pulpal exposure and the tooth is symptomless or with reversible pulpitis without any periapical pathologies, pulpotomy can be used to preserve radicular pulp and to treat inflammation and pain [2]. Pulpotomy can also be performed in teeth with either recently traumatic pulp exposure or mechanical pulp exposure [3]. Two common materials used for pulpotomy treatment are mineral trioxide aggregate (MTA) and calcium enriched mixture (CEM) cement [4, 5]. Some advantages of MTA are fast application, low cytotoxicity, biocompatibility, low microleakage, ability to set in the presence of blood or moisture, and antimicrobial properties [4, 6, 7]. CEM cement is biocompatible, tooth-colored, and hydrophilic (enabling it to set in a wet environment) [5].

Compared to teeth with healthy pulps, restored pulpotomized teeth are more susceptible to fracture. The fracture resistance of restored pulpotomized teeth is affected by the type of restorative material [8]. Different types of restorative materials have been used as final restoration in pulpotomized teeth [1]. Amalgam has good resistance against masticatory forces in the posterior teeth. However, cavity preparations for amalgam need to incorporate retentive features. This will finally weaken the tooth structure [9].

Pulpotomized teeth may also be restored with composite resins. Because of their adhesion to the tooth structure, composite resins usually do not need a mechanical undercut for retention [10]. Composite resins may also reinforce the remaining tooth structure due to their ability to transmit and distribute functional stresses through the bonding interface [8]. However, one of the major shortcomings of conventional composite resins is polymerization shrinkage. Therefore, incremental placement of resin composites has been suggested to reduce polymerization shrinkage stress [11].

Recently, bulk-fill composite resins have been developed to simplify the placement of direct composite restorations. Bulk-fill composite resins can be cured effectively at a depth of at least 4 mm. They demonstrate a low polymerization shrinkage stress at the same time [12, 13]. Another material which can be used for the restoration of pulpotomized teeth is giomer. Giomer has prereacted glass ionomer fillers that have been incorporated into resin-based materials to maintain the clinical advantages of GICs and to solve their potential dehydration issues as well as poor aesthetics [14]. Recently, low and high viscosity bulk-fill giomers (Beautifil Bulk Flow, SHOFU, and Beautifil Bulk, SHOFU, Kyoto, Japan) have been introduced [15]. In a previous study, bulk-fill giomers exhibited a cure depth of 4.0 mm and the same flexural strength and modulus as bulk-fill resin composites [16].

To the best of the authors' knowledge, there are no published data about the fracture resistance of permanent teeth pulpotomized with MTA and CEM cement and restored with different restorative techniques. Therefore, this study aimed to evaluate the fracture resistance of pulpotomized premolars (treated with MTA or CEM cement) with MOD cavities restored with various restorative techniques. The null hypothesis was that the fracture resistance of pulpotomized permanent premolars (treated with MTA or CEM cement) would not be affected by the various restorative techniques.

## 2. Materials and Methods

The study protocol was approved by the Research and Ethics Committee of Shiraz University of Medical Sciences (Protocol #IR.SUMS.DENTAL.REC.1399.048). One hundred and eight intact, unrestored, noncarious human maxillary premolars extracted for periodontal or orthodontic reasons with similar dimensions (buccolingual width = 8.5–10 mm; mesiodistal width = 6.5–8 mm) determined with a digital caliper (Mitutoyo, Tokyo, Japan) were selected. A light microscope at ×20 magnification was used to check each tooth for any existing enamel cracks or fractures, and the teeth with enamel cracks or fractures were excluded and replaced by teeth free of cracks. Written informed consent was signed by the patients whose extracted teeth were used for this study. One blinded calibrated operator performed all the procedures of this experimental study. After removing calculus and soft tissue deposits from the selected teeth using a hand scaler (Gracey curette SG 17/18, HuFriedy, Chicago, IL, USA), they were stored in a 0.5% chloramine solution for 24 hours. Then, they were stored in physiological saline at 4°C for less than one month after extraction until use.

The teeth were embedded in acrylic resin blocks (Acropars, Marlik Co., Tehran, Iran) up to 1 mm below the cement-enamel junction (CEJ). The long axes of the mounted teeth were oriented parallel to those of the molds, and their facial and lingual cusps were in the same plane.

The teeth were randomly assigned to nine groups of 12 teeth each. The first group was considered as the negative control group in which the teeth were left intact without any cavity preparation. Class II mesioocclusodistal (MOD) cavities with a depth of 4.0 mm and flat floors without proximal steps were prepared in the other experimental groups using high-speed diamond burs (Diatech, Heerbrugg, Germany) that were replaced after every four preparations. The gingival floors of the cavities were located 1.0 mm above the CEJ. The buccolingual width of each cavity which was extended into the pulp chamber was half the intercuspal distance. All cavosurface margins were prepared at 90°, and the internal line angles were rounded. The facial and lingual walls of the MOD cavities were prepared parallel to each other. All measurements were performed using a digital caliper (Mitutoyo, Corp, Kawasaki, Japan). The pulp chamber roofs and pulp horns were completely removed, and the coronal pulp tissue was amputated to expose the canal orifices.

After the preparations of the MOD cavities in groups 2, 4, 6, and 8, tooth-colored MTA (ProRoot, Dentsply, Tulsa Dental Specialties, Tulsa, OK, USA) was prepared on a glass mixing slab according to the manufacturer's instructions. Approximately 3 mm of MTA was placed over the root canal orifices and gently adapted to the dentinal walls using a cotton pellet.

In groups 3, 5, 7, and 9, after preparing the MOD cavities, CEM cement (Bionique Dent, Tehran, Iran) powder and liquid were mixed and placed over root canal orifices in a 3 mm thick layer according to the manufacturer's instructions.

After placing a moistened cotton pellet directly over the MTA or CEM cement, the tooth was temporized with Cavite (Ariadent, Tehran, Iran) for one week. The appropriate thickness of the MTA or CEM cement was confirmed by taking a radiograph. After one week, the interim filling was removed and the complete setting of MTA or CEM cement was assessed.

The teeth in groups 2 and 3 (positive control groups) were not restored after placing MTA or CEM cement in MOD-prepared cavities.

A 2 mm thick layer of a conventional glass ionomer cement (CGIC; GC Fuji II, GC Corporation, Tokyo, Japan) was placed over the set MTA in group 4 and over the CEM cement in group 5 according to the manufacturer's instructions. After complete setting of CGIC, the teeth in groups 4 (MTA + CGIC + amalgam) and 5 (CEM + CGIC + amalgam) were restored with a high-copper amalgam (ANA2000, Nordiska Dental, Angelholm, Sweden) using universal metal matrix band in a Tofflemire matrix retainer (Tofflemire, GPC, India).

After placing and light curing (for 20 seconds) a 2 mm thick layer of a resin-modified glass ionomer cement (RMGIC; Fuji II LC, GC Corporation, Tokyo, Japan) over the set MTA in group 6 and over CEM cement in group 7 according to the manufacturer's instructions, the internal walls of the cavities were acid etched with phosphoric acid (PA) 37% (Scotchbond TM Universal Etchant, 3M ESPE) for 15 s, washed for 20 s, and then gently air-dried. Afterward, an etch-and-rinse adhesive bonding system (Adper Single Bond 2; 3M ESPE, St. Paul, MN, USA) was applied on the RMGIC and the prepared surfaces of the cavities. Then, it was light cured according to the manufacturer's instructions. A metal matrix band (Adapt Super Cap Matrices, Kerr, Switzerland) was placed around the tooth in the Tofflemire matrix retainer (Tofflemire, GPC, India) after adhesive polymerization. The prepared cavities in groups 6 and 7 were restored using a conventional composite resin (Filtek Z250, 3M ESPE) which was placed in horizontal increments of 2 mm layer thickness. The layer of conventional composite resin was cured for 20 s using a light curing unit (VIP Junior, Bisco, Schaumburg, IL, USA) at 600 mW/cm2. After matrix removal, the restorations were cured for additional 10 s from buccal and palatal sides.

After complete setting of MTA and CEM cement in groups 8 and 9, the prepared internal walls of the cavities were acid etched, and adhesive bonding was applied as in groups 6 and 7. Then, a 2 mm thick layer of a low viscosity bulk-fill giomer (Beautifil Bulk Flow, SHOFU, Kyoto, Japan) was placed over MTA or CEM cement and light cured for 20 s using the light curing unit at 600 mW/cm2. Then, a 2 mm thick layer of a high viscosity bulk-fill giomer (Beautifil Bulk, SHOFU) was placed over the cured low viscosity bulk-fill giomer according to the manufacturer's instructions and light cured for 20 s using the light curing unit at 600 mW/cm^2^. After matrix removal, the restorations were cured for additional 10 s from buccal and palatal sides. Finishing (using diamond finishing burs (Diatech Dental AC)) and polishing (with polishing discs (Soflex, 3M ESPE) and rubber points) of the restorations were performed using a high-speed handpiece under an air/water spray.

After one-day water storage in 100% humidity at 37°C, the specimens were placed into a universal testing machine (Instron Z020, Zwick Roell, Ulm, Germany) for fracture resistance testing. Then, they were loaded compressively at 1 mm/minute. An occlusal load was applied perpendicular to the long axis of the tooth using a steel sphere (8 mm in diameter) which was in contact with the occlusal slopes of buccal and palatal cusps. When fracture occurred, the load was recorded in Newtons (N). The fracture pattern of each specimen was assessed using a stereomicroscope (×40). The fractures limited to the coronal portion were characterized as “mode 1 fractures or restorable” and those that reached the root were considered as “mode 2 fractures or not restorable”.

The means and standard deviations of each experimental group were calculated. The normality of the data was assessed using the Kolmogorov–Smirnov test. The one-way analysis of variance (one-way ANOVA), followed by the Bonferroni post hoc test, was used for data analysis. The data analyses were performed using SPSS software version 17 (SPSS Inc., Chicago, USA). *p* values less than 0.05 were considered statistically significant.

## 3. Results

The mean fracture resistances (N) and standard deviations of the experimental groups are presented in Table 1. The one-way ANOVA revealed statistically significant differences in the mean fracture resistance values among the nine experimental groups (*p*=0.001). Bonferroni post hoc test was used for pairwise comparisons.

The fracture resistance of sound premolar teeth (group 1, negative control, 939.44 ± 114.03 N) was significantly higher than those of the other groups (*p* < 0.05). Group 2 (nonrestored teeth pulpotomized with MTA, 273.60 ± 45.15) had the lowest fracture resistance value. The fracture resistance of group 2 was statistically lower than those of all experimental groups (*p* values < 0.05) except groups 3, 4, and 5 (*p* values > 0.05). No statistically significant differences were found between nonrestored teeth pulpotomized with MTA or CEM cement (groups 2 and 3) and the teeth pulpotomized with CEM cement or MTA and restored with GIC + amalgam (groups 4 and 5) (*p* values > 0.05). Although the fracture resistances of the teeth restored with amalgam (groups 4 and 5) were lower than those of the teeth restored with conventional composite resin or bulk-fill giomer (groups 6, 7, 8, and 9), these differences were not statistically significant (*p* values > 0.05). Groups 6, 7, 8, and 9 showed statistically significantly higher fracture resistance values than group 2 (*p* values < 0.05). No statistically significant differences were found among groups 6, 7, 8, and 9 (*p* values < 0.05).

The frequency (%) of failure modes among the experimental groups has been illustrated in Table 2. More unrestorable fracture patterns were observed in all the prepared teeth than in the intact ones. The highest rate of mode 1 fracture (restorable fracture) was observed in group 1 followed by groups 8 and 9. In the groups restored with composite resins or giomers (groups 6, 7, 8, and 9), the frequency of mode 1 fracture (restorable fracture) was higher than that of mode 2 fracture (unrestorable fracture). In nonrestored teeth pulpotomized with MTA or CEM cement (groups 2 and 3) and in teeth pulpotomized with MTA or CEM cement restored with GIC + amalgam (groups 4 and 5), mode 2 fracture (unrestorable fracture) had a higher frequency than mode 1 fracture (restorable fracture).

## 4. Discussion

This study was conducted to evaluate the effects of various restorative techniques on the fracture resistance and fracture pattern of pulpotomized premolars treated with MTA or CEM cement. The null hypothesis was accepted as different restorative techniques resulted in statistically similar fracture resistances. According to the results of this study, although no significant differences were found in the fracture resistance of the teeth restored with amalgam or composite resins, the highest rate of mode 1 fracture (restorable fracture) was observed in intact teeth followed by pulpotomized teeth restored with bulk-fill giomers.

The effects of restorative techniques on the fracture resistance of the pulpotomized teeth were assessed in the present study. To simulate the clinical situation following pulpotomy treatment, Class II MOD cavities were prepared in this study. Maxillary premolars were selected for this experimental study because of their low crown volume and crown/root ratio which make them more susceptible to cusp fracture compared with other posterior teeth [17].

Generally, a deep and extended cavity resulting from the preparation of an endodontic access cavity reduces the dentin amount of cusps and ridges to a critical extent, removes the arched roof of the pulp chamber, and thus negatively affects the strength of the tooth [8]. Furthermore, tooth preparation performed during pulpotomy may potentially increase cuspal deflection as well as the possibility of cusp fracture during function [4]. Therefore, the restoration of pulpotomized immature teeth is a challenge for dentists [1]. Undoubtedly, an appropriate restorative technique which ensures the tooth function, maintains the tooth structure against fracture, and meets the aesthetic needs of the patients should be selected [18]. The results of this study showed that the fracture resistance of the pulpotomized teeth was lower than that of the intact teeth which is due to the loss of tooth structure in pulpotomized teeth. This result is in line with previous studies which demonstrated that the fracture resistance of the teeth decreased after cavity preparation [18, 19].

Amalgam is an inexpensive and easy-to-handle material which can be used to restore pulpotomized teeth. Amalgam has good longevity and clinical characteristics such as low technique sensitivity and self-sealing ability [11]. However, it does not bond to the tooth structure, and thus cavity preparations incorporating retentive features are needed [9]. The results of the present study showed that amalgam could not enhance the fracture resistance of the pulpotomized teeth compared to that of prepared nonrestored teeth. This can be justified by the fact that amalgam is not capable of reinforcing the remaining tooth structure [9].

Other materials which have been widely used in the restoration of pulpotomized teeth are composite resins. Composite resins can reinforce the weakened remaining tooth structure due to their micromechanical bonding to the remaining tooth structure [20]. However, one of the limitations of conventional composite resins is polymerization shrinkage. To reduce the polymerization stress of composite materials, an incremental composite placement technique has been recommended. Although the incremental filling technique reduces the polymerization shrinkage stress of conventional composite resin restorations, it is time-consuming, increases the probable risk of contamination between layers, and may lead to void formation in composite resin restorations [12]. To overcome these limitations, bulk-fill composite resins have been introduced which can be placed and light cured in a single step [13].

Another material which can be used for the restoration of pulpotomized teeth is giomer. The primary pulpotomized molars restored with giomer demonstrated a higher fracture strength than amalgam and GIC in a previous study [21]. Recently, low and high viscosity bulk-fill giomers (Beautifil Bulk Flow, SHOFU, and Beautifil Bulk, SHOFU, Kyoto, Japan) have been introduced [15]. It has been reported that flowable composite resins may act as a stress breaker, increase flexibility, and work against fractures [22, 23]. Therefore, in the present study, a 2 mm thick layer of a high viscosity bulk-fill giomer (Beautifil Bulk, SHOFU) was placed over the low viscosity bulk-fill giomer.

The results of the present study revealed that both conventional composite resin and bulk-fill giomer had a reinforcing effect on the weakened tooth structure to some extent. However, no statistically significant difference was found between the fracture resistances of the conventional composite resin restorations and those of bulk-fill giomers. A previous study demonstrated that the flexural strength and modulus of bulk-fill giomers were comparable to those of composite resins, justifying the findings of the present study [16]. Further long-term studies with larger sample sizes and different restorative groups may reveal the probable differences between bulk-fill giomers and conventional composite resins.

In the present study, no statistically significant differences were found in the fracture strength of teeth restored with amalgam, conventional composite resin, and bulk-fill giomers. Some studies have demonstrated that composite restorations reinforce the tooth better than amalgam [24, 25], while others have found no differences [19, 26]. The differences in the findings of the previous studies can be attributed to the differences in sample sizes, tooth preparations, and fracture strength test methods.

In the present study, two types of materials (including MTA and CEM cement) were used for pulpotomy treatment of the teeth. A similar performance in the pulpotomy of immature caries-exposed permanent molars has been previously demonstrated for CEM cement and MTA [27]. According to the results of the present study, the type of material used for pulpotomy did not affect the fracture resistance of the restored teeth, and CEM cement and MTA demonstrated comparable results. Therefore, both MTA and CEM cement can be effectively used for pulpotomy treatment without compromising the fracture resistance of the teeth.

In this study, although no differences were found among the fracture resistance values of amalgam, conventional composite resin, and bulk-fill giomer restorations, the frequencies of the fracture modes were different among the experimental groups. Most of the fractures that occurred in the pulpotomized teeth restored with conventional composite resins or bulk-fill giomers were restorable. However, most of the pulpotomized teeth restored with amalgam in the present study exhibited unrestorable fractures. Among the restored teeth in this study, the highest rates of restorable fractures occurred in the teeth restored with bulk-fill giomers. The higher rate of restorable fracture in the teeth restored with conventional composite resins and bulk-fill giomers can be attributed to the mechanical interlocking of resin with dentin and hybrid layer formation. This micromechanical bonding results in the transmission and distribution of functional stresses through the restorative material-tooth interface and finally reinforces the weakened tooth structure [28].

The method of occlusal loading during the fracture strength test is an important factor affecting the results of the test. Axial forces applied to the center of the occlusal surface were used in this study. However, lateral forces and fatigue loading are also present during function in the oral cavity. The speed and direction of the applied load were constant in the current study, and the load was constantly increased until fracture occurred. However, masticatory forces usually have different directions, variable speeds, and longer periods [29]. The restorative material should have the ability to withstand different types of forces (such as excessive functional and parafunctional forces) in the oral cavity without fracture [30–32]. Therefore, it is recommended that the findings of this study be verified in future studies using the chewing simulation device before fracture testing.

This study had some limitations. First, the current study was an in vitro study and the fracture test was performed 24 hours after restoration. Future long-term studies considering the effects of chemical, thermal, and physical stresses in the oral cavity should also be conducted to clarify the results of the present study. Additionally, a continually increasing load was applied to the teeth in this study for the fracture test which is not a typical loading that occurs clinically. Further clinical investigations are needed to verify the results of this in vitro study.

## 5. Conclusion

According to the results of the present study, MOD cavity preparations during pulpotomy treatment reduced the fracture resistance of the teeth. The fracture resistances of all of the restoration groups were higher than that of the prepared-only group and lower than that of the intact teeth group. Therefore, none of the tested restoration techniques was able to restore completely the fracture resistance of the pulpotomized teeth with MOD-prepared cavities. No significant differences were found among the fracture resistances of the restored teeth. However, considering the fracture modes, bulk-fill giomers followed by conventional composite resins were better able to prevent unfavorable fractures than amalgam. Therefore, they seem to be more reliable for the restoration of pulpotomized teeth with MOD cavities.

## Figures and Tables

**Table 1:** The mean fracture resistances (N) and standard deviations of the experimental groups.

Groups	Restoration type	Mean ± standard deviation
Group 1	Intact teeth	939.44 ± 114.03^A^
Group 2	Nonrestored teeth pulpotomized with MTA	273.60 ± 45.15^B^
Group 3	Nonrestored teeth pulpotomized with CEM cement	332.69 ± 33.51^B^
Group 4	MTA pulpotomized teeth restored with GIC + amalgam	382.40 ± 54.08^BC^
Group 5	CEM cement pulpotomized teeth restored with GIC + amalgam	331.075 ± 60.546^BC^
Group 6	MTA pulpotomized teeth restored with RMGIC + conventional composite resin	431.40 ± 45.92^C^
Group 7	CEM cement pulpotomized teeth restored with RMGIC + conventional composite resin	418.96 ± 141.99^C^
Group 8	MTA pulpotomized teeth restored with a low viscosity bulk-fill giomer + a high viscosity bulk-fill giomer	444.60 ± 80.66^C^
Group 9	CEM cement pulpotomized teeth restored with a low viscosity bulk-fill giomer + a high viscosity bulk-fill giomer	394.65 ± 52.74^C^

Within column, mean values with different uppercase superscript letters indicate statistically significant differences at a significance level of 0.05 (Bonferroni post hoc test).

**Table 2:** The frequency (%) of failure modes among the experimental groups (*n* = 12).

Groups	Restoration type	Mode 1 fracture (restorable)	Mode 2 fracture (unrestorable)
Group 1	Intact teeth	11 (91%)	1 (9%)
Group 2	Nonrestored teeth pulpotomized with MTA	4 (33%)	8 (67%)
Group 3	Nonrestored teeth pulpotomized with CEM cement	3 (25%)	9 (75%)
Group 4	MTA pulpotomized teeth restored with GIC + amalgam	5 (41%)	7 (59%)
Group 5	CEM cement pulpotomized teeth restored with GIC + amalgam	4 (33%)	8 (67%)
Group 6	MTA pulpotomized teeth restored with RMGIC + conventional composite resin	7 (58%)	5 (42%)
Group 7	CEM cement pulpotomized teeth restored with RMGIC + conventional composite resin	7 (58%)	5 (42%)
Group 8	MTA pulpotomized teeth restored with a low viscosity bulk-fill giomer + a high viscosity bulk-fill giomer	8 (66%)	4 (34%)
Group 9	CEM cement pulpotomized teeth restored with a low viscosity bulk-fill giomer + a high viscosity bulk-fill giomer	8 (66%)	4 (34%)

## Data Availability

The data that support the findings of this study are available upon request from the corresponding author.

## Abbreviations

MTA: Mineral trioxide aggregate
CEM cement: Calcium enriched mixture (CEM) cement
GIC: Glass ionomer cement
RMGIC: Resin-modified glass ionomer cement.
